# Comorbidity Between Anti-GAD65 Autoimmune Encephalitis and Behavioral Variant Frontotemporal Dementia: A Case Report

**DOI:** 10.3390/reports9020133

**Published:** 2026-04-26

**Authors:** Sergiu Băjan, Anastasia Kateryna Sikora-Medvid, Simona Claudia Tămășan, Alina Murariu, Virgil Radu Enătescu

**Affiliations:** 1 Doctoral School, Faculty of General Medicine, “Victor Babeș” University of Medicine and Pharmacy Timisoara, 300041 Timișoara, Romania; 2 ”Eduard Pamfil” Psychiatric Clinic, Timisoara County Emergency Clinical Hospital, 300425 Timisoara, Romania; enatescu.virgil@umft.ro; 3 Department of Psychiatry, Addictions and Medical Psychology, Ivano-Frankivsk National Medical University, 76018 Ivano-Frankvisk, Ukraine; asikora@drewnica.pl; 4 Mazovian Specialist Hospital “Drewnica”, 05-091 Ząbki, Poland; 5 Department of Psychiatry, English Division, Medical University of Warsaw, 02-091 Warszawa, Poland; 6 Liaison Psychiatry, “Pius Brînzeu” Emergency Clinical County Hospital, 300425 Timișoara, Romania; simona.tamasan@umft.ro; 7 Center for Modeling Biological asystems and Data Analysis, "Victor Babes" University of Medicine and Pharmacy, 300041 Timisoara, Romania; 8 Neurology II Department, “Pius Brînzeu” Emergency Clinical County Hospital, 300425 Timișoara, Romania; alyna.murariu@yahoo.com; 9 Department of Neurosciences, Discipline of Psychiatry, “Victor Babes” University of Medicine and Pharmacy, Eftimie Murgu Square 2, 300041 Timișoara, Romania

**Keywords:** encephalitis, anti-GAD65 antibodies, frontotemporal dementia, case report, plasmapheresis

## Abstract

**Background and clinical significance**: Autoimmune encephalitis (AE) is an inflammatory brain disorder that manifests through a diverse, unspecific range of neuropsychiatric symptoms. When AE occurs alongside a primary neurodegenerative disorder, the shared symptoms can create a mixed clinical profile, making diagnosis more difficult and potentially postponing effective management and treatment. **Case presentation**: We describe the case of a 58-year-old female with a one-year history of progressive behavioral and personality changes who presented a subacute confusional state, psychomotor retardation alternating with psychomotor agitation, apathy, visual hallucinations, and motor symptoms. Examination revealed Parkinsonian symptoms and frontal lobe signs. Neuroimaging showed frontotemporal atrophy, while cerebrospinal fluid analysis excluded infection but demonstrated elevated phosphorylated tau, supporting an underlying neurodegenerative process. An electroencephalogram revealed asymmetric temporal slowing without overt epileptiform activity. An initial diagnosis of behavioral variant frontotemporal dementia (bvFTD) was established. Due to rapid clinical deterioration and fluctuating cognition, autoimmune testing was expanded to a full antibody panel, which identified elevated serum anti-glutamic acid decarboxylase 65 (anti-GAD65) antibodies (60 UI/mL, reference range 0–5 UI/mL), establishing a possible coexisting diagnosis of anti-GAD65 autoimmune encephalitis. Initial treatment with intravenous immunoglobulin produced minimal improvement; however, therapeutic plasma exchange led to the remission of psychosis and significant improvement in rigidity, bradykinesia, and attention, with modest amelioration in global cognition. **Conclusions**: This case highlights the diagnostic challenges posed by overlapping AE and bvFTD clinical pictures, especially when neurodegenerative features obscure an underlying autoimmune process. Early, panel-based neural antibody testing—and consideration of AE even in patients already diagnosed with a major neurocognitive disorder—is critical for avoiding delays in immunotherapy. Prompt recognition and treatment of AE may substantially improve clinical outcomes, even in complex cases with suspected overlap.

## 1. Introduction and Clinical Significance

Autoimmune encephalitis is a rare neurological disorder (1–5 per million person-years [1]) in which the immune system targets neuronal proteins, causing brain network dysfunction [2]. One uncommon subtype is anti-glutamic acid decarboxylase autoimmune encephalitis (GAD-AE), characterized by the production of autoantibodies targeting GAD65, inhibiting the synthesis of gamma-aminobutyric acid (GABA), which leads to an excessive neuronal activation [3]. Consequently, patients may present diverse clinical manifestations such as limbic or extralimbic encephalitis (LE) with subacute cognitive and/or psychiatric symptoms, stiff-person syndrome (SPS), cerebellar ataxia, and epilepsy [3,4].

The incidence of anti-GAD65 antibodies is estimated at 1.9 per 100,000 person-years in the general population [5]. To our knowledge, data regarding the prevalence of anti-GAD-AE is not available in the current literature. Risk factors include female sex (female-to-male ratio ~9:1) [6], coexisting autoimmune disorders (e.g., type 1 diabetes, thyroiditis), and, rarely, neoplasia (e.g., thymoma or small-cell lung cancer) [7].

Frontotemporal dementia (FTD) encompasses a group of neurodegenerative disorders characterized by progressive atrophy of the frontal and temporal lobes, leading to changes in behavior, executive function, and language [8]. This spectrum includes behavioral variant FTD (bvFTD) as well as the nonfluent and semantic variants of primary progressive aphasia, each potentially accompanied by amyotrophic lateral sclerosis [9]. The bvFTD, the most common subtype accounting for 50–70% of FTD cases, is defined by early and prominent behavioral changes, including apathy, disinhibition, loss of empathy, and impaired social cognition, often associated with psychotic symptoms [8,10].

Autoimmune encephalitis can mimic neurodegenerative dementias—including bvFTD—leading to delayed recognition unless “red flags” (subacute course, seizures, inflammatory cerebrospinal fluid (CSF)/magnetic resonance imaging (MRI), fluctuation) are sought [11]. The overlapping symptoms consist of deterioration in social conduct and personality change (cardinal features of FTD) along with other features commonly seen in FTD, such as executive dysfunction, decline in self-care, stereotyped behavior, and altered speech output but with relative preservation of memory [12]. Citing literature data, approximately 25% of AE cases were misdiagnosed [13].

The current paper showcases a rare and diagnostically challenging case: an initial diagnosis of bvFTD, later found to have possible coexistence with anti-GAD65 AE. The autoimmune component was suspected based on elevated serum antibody titers, with partial clinical improvement observed following plasmapheresis. This case aims to raise awareness among clinicians about this unusual association, emphasizing the importance of AE antibody screening in patients with a dementia-like clinical picture.

## 2. Case Presentation

A 58-year-old Caucasian woman, a physician by profession, with no documented pre-existing medical conditions presented to the emergency department (ED) with apparently acute onset confusion with cognitive impairment (time and place disorientation, short-term memory loss, perseverations), marked psychomotor retardation, apathy, and transitory visual hallucinations. Given the fact that she has lived alone for the past two years, a reliable and comprehensive history of recent behavior and a precise onset of symptoms were difficult to obtain at the time. Eventually, a follow-up history-taking involving the family members and the patient’s co-workers revealed behavioral and personality changes during the past year, which have been worsening in the recent period. Furthermore, family history also disclosed multiple maternal relatives being affected with major neurocognitive disorders.

Vitals on admission were: blood pressure of 127/76 mmHG, heart rate of 76 beats per minute, respiratory rate of 17 breaths per minute, and body temperature of 36.3 °C. Peripheral oxygen saturation on ambient air was 97%.

The neurological examination showed bradykinesia, symmetric extrapyramidal rigidity, camptocormia and a positive Romberg test. Convergence insufficiency of ocular globes and fine spontaneous nystagmus were noticed. The Hoffman sign, the Babinski sign, and brisk deep tendon reflexes were present on the left side. The rest of the physical examination was within normal limits.

**Figure 1:**
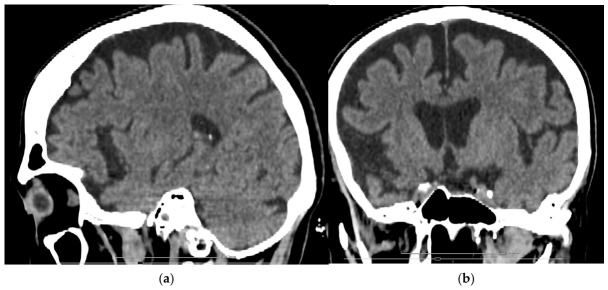
Brain CT findings: (**a**) sagittal view demonstrating predominant frontal and anterior temporal cortical atrophy; (**b**) coronal view showing bilateral frontotemporal volume loss with associated enlargement of the frontal horns/temporal horns.

To exclude acute tumoral or vascular pathology, a native brain computed tomography (CT) scan was performed in the ER, which showed a symmetrically enlarged ventricular system with reduced gyral volume and widened frontotemporal sulci (Figure 1).

**Figure 2:**
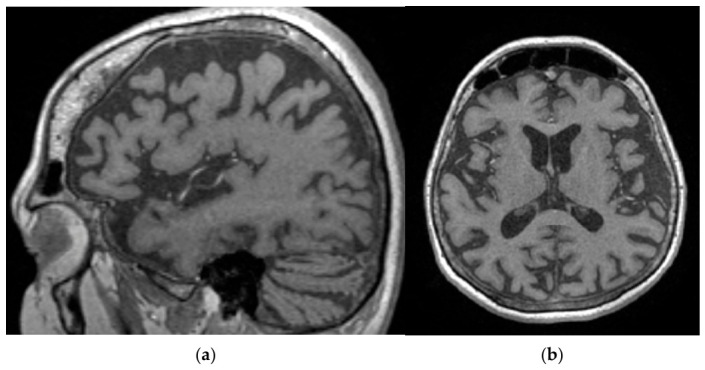
MRI scan: (**a**) and (**b**) bilateral frontotemporal volume loss with secondary ventriculomegaly.

Under clinical suspicion of acute encephalitis, the patient was hospitalized in the neurology department for a comprehensive diagnostic workup and treatment planning. The initial blood analyses showed elevated values of inflammatory markers (C-reactive protein (CRP), erythrocyte sedimentation rate (ESR), fibrinogen), but with no relevant modifications of procalcitonin or full blood-count (FBC). Serum blood glucose was within normal range at all measurements. A brain MRI with intravenous (IV) contrast agent was made, which showed no pathological contrast enhancement (Figure 2). The patient also underwent thoracic, abdominal, and pelvic native + IV contrast CT, with no abnormalities found.

A lumbar puncture with CSF analysis was undertaken to rule out an infectious etiology. Results demonstrated mild proteinorachia and glycorrhachia, with no other cytological features suggestive of infection. CSF cultures were negative for bacteria and fungi. Phosphorylated tau protein levels were elevated.

**Figure 3:**
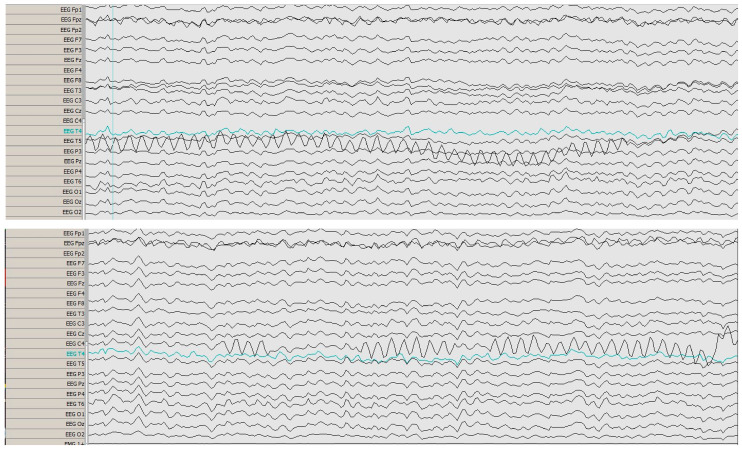
EEG showing atypical, asymmetrical sharp Theta waves in the temporal area, suggestive of encephalopathy.

The electroencephalogram (EEG) was abnormal, with atypical, asymmetrical sharp Theta waves in the temporal area; no clearly defined epileptogenic foci were identified (Figure 3).

Considering the possibility of an autoimmune etiology, the patient received probatory treatment with IV administration of immunoglobulin G (IgG), 20 g per day for five days, with no apparent clinical improvement. Further blood analysis revealed the presence of anti-VCA immunoglobulin M for Epstein–Barr virus, suggesting an acute or subacute viral infection. Considering the possibility of a viral encephalitis, the patient received IV Acyclovir, 500 mg q6, for 10 days, with an initial slight favorable clinical response, but with subsequent deterioration. Simultaneously, the patient was treated with medications including valproic acid (500 mg q12h), memantine (5 mg qd), quetiapine (50 mg bid), venlafaxine (37.5 mg qd, following liaison psychiatry consult), and levodopa and carbidopa combination (68.75 mg q8h), with poor clinical results. During hospitalization, the patient’s symptomatology had a fluctuating course with subsequent decline, marked by psychomotor agitation with dromomania, exacerbation of visual hallucinations, deterioration of executive functions, and worsening of the perseverative and stereotyped behavior.

Given the clinical presentation, paraclinical and neuroimaging investigations, and the subsequent anamnestic data, an initial diagnosis of bvFTD was strongly considered. The patient was transferred to the psychiatric department for further management.

Upon admission to the Psychiatric Department, a Mental State Examination (MSE) was performed. The MSE revealed a disheveled appearance, with unkempt clothing and poor personal hygiene, consistent with apathy and reduced self-care. There was reduced spontaneous activity with occasional motor stereotypies and utilization behavior (grasping/examining objects unprompted). Eye contact was minimal. Intermittent outbursts of psychomotor agitation were noted. Speech was slow and quiet, with reduced fluency and increased response latency. Affect was blunted, with restricted range—minimal emotional reactivity and frustration during cognitive tasks. Thought process was impoverished, with perseverations and concrete thinking on abstract tasks. Assessment of thought content proved challenging, with no delusions, suicidal ideation, or obsessions elicited. Visual zoomorphic hallucinations were present. The patient was disoriented to time and place, oriented to person. She showed deficits in executive functions (planning, prioritizing and sequencing of multitasking activities, organization and new information learning skills, task initiation, sequencing, problem solving). Working and short-term memory were relatively spared. Attention was impaired. Visuospatial and visuoconstructional skills were reduced due to planning deficits. Insight was absent.

The patient underwent cognitive evaluation using the Mini-Mental State Examination (MMSE) and the Montreal Cognitive Assessment (MoCA). The MMSE showed a score of 11/30 (maximum possible score correlated with patient’s age and level of education). The MoCA showed a score of 3/30.

Over the course of the admission, the patient received psychopharmacological treatment with second-generation antipsychotics: Clozapine (25 mg qd), subsequently replaced with Risperidone (2 mg q8h), Quetiapine (200 mg qd), and Tiapride (50 mg q12h); an NMDA receptor antagonist: memantine (20 mg qdpm); an acetylcholinesterase inhibitor: donepezil (5 mg qdpm); an anticonvulsant: gabapentine (400 mg qd);an anticholinergic: trihexyphenidil (1 mg q12h); a dopamine receptor agonist: Levodopa in combination with Carbidopa (68.75 mg q8h); and a neurotrophic agent: porcine brain-derivate hydrolysate (10 mL qdam, for ten days).

Given the unfavorable clinical course with fluctuation of symptoms, a complete AE antibody panel was requested. The AE evaluation revealed laboratory-positive autoantibodies to GAD65, with a serum level of 60 UI/mL (reference range 0–5 UI/mL). No other antibodies were present. Considering the elevated anti-GAD65 antibody titers and the acute aggravation and fluctuation of the recent clinical picture, superimposed anti-GAD65 AE was deemed the most probable diagnosis. The patient was re-transferred to the Neurology Department with a recommendation to initiate therapeutic plasma exchange (TPE), given that the symptoms proved refractory to IV IgG treatment.

A course of five TPE sessions was planned, performed on every other day. Enoxaparin sodium (0.4 mL qd) was administered to prevent thrombo-embolic complications. After the second TPE session, a favorable clinical response was observed, with complete remission of positive psychotic symptoms and improvement in cognition and motor function (gait, posture, rigidity, praxia, bradykinesia). Following the fifth and final session, the neurocognitive tests were re-applied, with improvement on both scales. The MMSE score was 16/30, with a 5-point increase compared to the initial score, and the MoCA score was 6/30, with a 3-point increase compared to the initial score.

The patient was discharged, with recommendations of psychopharmacological treatment consisting of risperidone (1 mg q8h), memantine (20 mg qdpm), donepezil (5 mg qdpm), tryhexyphenidil (1 mg q12h), levodopa + carbidopa (137.5 mg q6h), and follow-up neurological and psychiatric evaluation after 1 month.

At discharge, the patient was stable, with marked improvement in motor function and with full remission of psychotic elements. However, the clinical improvement was incomplete, with cognitive functions only slightly ameliorated, the patient needing continuous care and supervision by a caregiver. All the evolution of the patient is summarized in Table 1.

## 3. Discussion

The current paper presents the case of a 58-year-old female, with no known medical history, who presented in the ED with subacute/acute onset altered mental status and psychotic symptoms. Additionally, the patient’s behavior and personality progressively deteriorated over the course of the previous year. The symptomatology worsened in the recent period prior to the ED presentation. The patient presented frontotemporal atrophy on neuroimaging examination (CT/MRI), as well as positive anti-GAD65 antibodies in plasma. Support for active EBV encephalitis was limited to serum anti-VCA IgM, while there was no CSF virological confirmation, the response to acyclovir was only slight and transient, and the subsequent clinical course was more consistent with an autoimmune process. The symptomatology was refractory to psychopharmacological (anti-dementia and antipsychotic drugs) and IV IgG therapies, but improved partially after one course of five plasmapheresis sessions.

### 3.1. Anti-GAD65 Autoimmune Encephalitis

Encephalitis represents the inflammation of the brain parenchyma. The most commonly affected areas are the frontal and temporal lobes, basal ganglia, cerebellum, thalamus, and insula [14]. The principal etiologies of encephalitis are infectious and autoimmune. The prevalence of both causes is roughly equivalent (11.6/100,000 person years for infectious and 13.7/100,000 person years for autoimmune) [5]. Autoimmune encephalitis encompasses three known immunologic patterns, consisting of cell-surface protein antibodies, synaptic receptor antibodies, and antibodies against intracellular antigens, with anti-GAD65 antibodies being included in the latter category [14]. GAD is an enzyme that catalyzes glutamate to produce the neurotransmitter GABA. Two isoforms are recognized, specifically GAD65 and GAD67, classified by their molecular weights and characterized by distinct localization and functions (GAD67 is predominantly found in neuronal cytoplasm, whilst GAD65 is found in presynaptic terminals). Both are expressed in GABAergic neurons of the central nervous system (CNS), with GAD65 being the principal autoantigen of clinical interest given its higher autoantigenicity [15]. Anti-GAD65 antibodies block this enzyme, therefore interfering with its synthesis and affecting the inhibitory GABAergic neurocircuits. As a result, a state of CNS hyperexcitability is induced, leading to the manifestation of neuropsychiatric symptomatology [4,16].

The mechanism of GAD antibody development may be paraneoplastic or autoimmune. The autoimmune theory claims that, although GAD is an intracellular protein, it may be exposed to extracellular humoral immunity during the exocytosis of GABAergic neurons; therefore, antibodies could bind to the cell surface and be directly pathogenic [17]. Antibodies may also be detected in conditions including (but not limited to) type 1 diabetes mellitus, autoimmune thyroid disease, and pernicious anemia [16]. They may also coexist with other autoimmune conditions such as Addison disease, vitiligo, and premature ovarian failure [15,16]. Within neurology, anti-GAD65 antibodies are classically linked not only to encephalitis but also to stiff-person syndrome, cerebellar ataxia, and epilepsy, and their clinical interpretation depends heavily on the syndrome, antibody level, and CSF findings [15,16]. In the current case, no neoplastic or autoimmune diseases commonly associated with positive anti-GAD65 antibodies were found.

### 3.2. Frontotemporal Dementia

FTD is recognized as one of the leading causes of early-onset dementia and typically presents in people in their late fifties. According to a study by Knopman et al. [18], about 10% of FTD cases are present in patients aged less than 45 years, and about 60% of FTD cases present in those aged between 45 and 64 years.

The pooled crude incidence for FTD was 2.28 (95% CI, 1.55–3.36) per 100,000 person-years, and the prevalence was 9.17 per 100,000 people. The bv-FTD pooled crude incidence was 1.20 per 100 000 person-years, and the prevalence, 9.74 per 100,000 people [19].

FTD is a neurodegenerative disorder that primarily affects the frontal and temporal lobes and leads to distinct clinical syndromes. FTD is classified into three clinical variants: bvFTD, which is characterized by early and significant changes in behavior, emotion, personality, and executive control; nonfluent-variant PPA (nfvPPA), which is characterized by progressive deficits in speech, grammar, and word output; and semantic-variant PPA (svPPA), which is a progressive disorder of semantic knowledge and naming [20]. These clinical syndromes are closely linked to specific molecular pathologies that affect the frontal and temporal lobes [21].

In bvFTD, most behavioral symptoms are caused by disruptions to the brain networks involved in emotions and social functions, including the medial orbitofrontal cortex, anterior temporal lobe, and anterior insula, as these regions match those affected by the pattern of cerebral atrophy characteristic of bvFTD [22,23]. Mutations have currently been associated with bvFTD in genes encoding multiple proteins, with the C9ORF72 (chromosome 9 open reading frame 72) mutation being the most common clinical associated phenotype. In total, 30 to 50% of patients with C9ORF72 expansions present with hallucinations and delusions [24,25].

### 3.3. Symptomatology

Clinical symptoms in AE can begin in a subacute manner (symptom onset in less than 3 months), or, on the contrary, the symptoms can appear progressively, sometimes mimicking the onset of a neurodegenerative disease [26,27].

The most common GAD antibody-related neurological phenotype is SPS, followed by cerebellar ataxia, refractory epilepsy, limbic encephalitis, and extralimbic encephalitis [28], in line with the previously reported literature [3]. Furthermore, autoimmune encephalopathy can present broad and undefined/unspecific clinical phenotypes, including manifestations that can resemble dementia [11]. Our patient presented a symptomatology consisting of a worsening of an already documented cognitive decline and a mixture of psychotic and behavioral symptoms, representing a phenotype close to the LE spectrum.

Similar to other types of LE, anti-GAD associated LE is characterized by the subacute development of deficits in working memory, paranoid symptoms, hallucinations, irritability, affective symptoms including emotional instability, confusion, behavioral changes, and epileptic seizures with leading temporal semiology [29,30]. Patients with GAD-associated LE often present with motor symptoms, including increased muscle tone, rigidity, bradykinesia, ataxic gait, catatonia, dyskinesias, and dystonic posturing.

Clinical presentation of bvFTD consists of progressive development of behavioral and personality changes, with a progressive decline in social cognition and executive abilities. The most pronounced behavioral symptoms of bvFTD are disinhibition (inappropriate or offensive behavior, excessive jocularity, exaggerated emotional display, impulsivity, inappropriate sexual remarks, lack of embarrassment) (73–98%), loss of empathy, lack of emotional insight, social coldness (49–78%), personal neglect, neglect of hygiene (83–92%), and behavioral stereotypies (95%), which typically precede any obvious cognitive impairment [8]. Patients with bvFTD tend to show deficits in executive function (with emphasis on ventromedial prefrontal cortex impairment, such as mental rigidity, inattention, disorganization, impaired decision making, error sensitivity, and verbal fluency). Moreover, in patients with FTD syndromes, behavioral and cognitive symptoms can overlap with motor symptoms, such as Parkinsonism, including bradykinesia, rigidity, tremor, and postural instability. Early Parkinsonism is observed in 16% of FTD patients, with bradykinesia being the most common manifestation in 84% of patients, followed by Parkinsonian gait (71%), rigidity, postural instability (35%), and resting tremor (6.5%) [31,32]. Patients with bvFTD caused by pathogenic expansions or mutations of the C9orf72 gene present with positive psychotic symptoms such as hallucinations and delusions in 50% of cases [24].

As shown, the clinical profiles of AE and bvFTD overlap: both can present with new behavioral changes, executive dysfunction, and psychiatric symptoms that evolve over weeks to months, often prompting an initial diagnosis of neurodegenerative dementia.

### 3.4. Diagnostic Criteria and Differential Diagnosis

#### 3.4.1. Autoimmune Encephalitis Diagnostic Approach

Diagnostic criteria for AE have been developed [33] that may be used to select patients for prompt treatment with immunotherapy after infection has been excluded, even before neural antibody tests have been confirmed [34]. The detection of specific autoantibodies establishes a definitive diagnosis of autoimmune encephalitis, identifies immunological subtypes of LE, and assists in the differential diagnosis of atypical clinical cases. Some authors suggest that the existing diagnostic criteria for autoimmune encephalitis are too reliant on antibody testing, as it is not readily accessible in many institutions and results can take several days or weeks to obtain [33]. However, the diagnosis of definite AE greatly depends on the results of autoantibody tests, especially for patients who do not present with well-defined syndromes. CSF analysis provides similar specificity, but worse sensitivity for GAD65 antibody levels [35].

According to Graus et al. [33], diagnosis for possible autoimmune encephalitis can be made when the following criteria have been met: subacute onset (rapid progression of less than 3 months) of working memory deficits, altered mental status (including altered level of consciousness, lethargy, or personality change), or psychiatric symptoms; at least one of the following: new focal CNS findings, seizures not explained by a previously known seizure disorder, CSF pleocytosis (white blood cell count of more than five cells/mm) or MRI features suggestive of encephalitis, and reasonable exclusion of alternative causes.

Positive diagnosis of AE was considered in our case due to the presence of a worsening cognitive function in the recent period (weeks/months), the acute onset of psychomotor agitation alternating with psychomotor retardation, and the presence of psychotic positive and negative symptoms, with new focal frontal and pyramidal neurological signs present. Moreover, paraclinical results supported this diagnosis (elevated inflammatory markers on laboratory results, EEG changes suggestive of encephalitis, positive anti-GAD autoantibodies on antineuronal autoantibody panel). Anti-GAD65 antibody titers were only modestly elevated in our case, but this finding should be interpreted in the context of prior IVIG treatment.

#### 3.4.2. bvFTD Diagnostic Approach

According to DSM-V, the diagnostic criteria for major neurocognitive disorder consist of: significant progressive decline in one or more cognitive domains (such as executive function, attention, memory, language, motor, or social cognition), based on a substantial impairment in cognitive performance, preferably documented by standardized neuropsychological testing or another quantified clinical assessment; said cognitive deficits interfere with independence in everyday activities; and the cognitive deficits do not occur exclusively in the context of delirium, and are not better explained by another medical disorder (major depressive disorder, schizophrenia, etc.). Major cognitive disorders are primarily subtyped according to the known or presumed pathological entity underlying the cognitive decline. These subtypes are distinguished by a combination of time course, characteristic domains affected, and associated symptoms.

For a bvFTD diagnosis, in addition to the criteria for major neurocognitive disorder, the following criteria must be met: insidious onset and gradual progression; prominent decline in social cognition and/or executive behavior; the presence of three or more of the following behavioral features: behavioral disinhibition, apathy, loss of empathy, perseverative/ritualistic behavior, and hyperorality/dietary changes; relative sparing of learning and memory and perceptual-motor function; the disturbance must not be better explained by cerebrovascular disease, another neurodegenerative disease, the effects of a substance, or another mental, neurological, or systemic disorder. The diagnosis of bvFTD is categorized as possible, probable, or definite according to the severity of cognitive and functional impairment. If there is supporting evidence from genetic mutations or neuroimaging (distinctive atrophy or reduced activity in frontotemporal regions on structural or functional imaging), the diagnosis is described as probable; otherwise it is designated as possible [8]. Definite bvFTD includes histopathologic evidence of frontotemporal lobe degeneration on biopsy or post-mortem [36].

Our patient presented a progressive, insidious cognitive decline in the past two years, associated with personality changes, including apathy, perseverative behavior, and an initial relative sparing of learning and memory (data collected from patient’s co-workers, as the patient initially retained a somewhat stable professional functionality). The sudden, rapid worsening of cognitive impairment with affected memory and visuospatial skills, with marked impairment in daily social and professional activities and the presence of anti-GAD65 titers in the patient’s serum, made us consider co-pathology with AE. Considering the already-present, steady cognitive decline, a diagnosis of autoimmune dementia was ruled out.

### 3.5. Treatment

Since no FDA-approved drugs exist for treating FTD, the primary emphasis lies on managing symptoms. Behavioral symptoms, such as irritability, aggressiveness, and disinhibition, can be improved with mood stabilizers or low doses of atypical antipsychotics, with the latter also being useful if psychotic symptoms (hallucinations, delusions) are present [8,37]. Moreover, there was a study that showed that patients with FTD undergoing treatment with memantine presented with a lower score on frontal behavior inventory (FBI) [38]. Treatment of cognitive symptoms has been relatively ineffective [37], with risperidone showing a positive effect even if no controlled studies have been conducted. Non-pharmacological interventions and strategies are useful, such as speech and cognitive therapies (such as non-verbal communication) and behavioral interventions, applied by the primary caregiver [9].

Similar to other autoimmune conditions, the treatment of GAD-associated neurological syndromes has not yet been codified/standardized, and the schemes of immune therapy are highly dependent on the experience of individual centers and physicians. The first-line treatment of LE relies on the administration of IVIG, high-dose intravenous corticosteroids, or TPE, alone or in combination, followed by a rapid switch to rituximab or cyclophosphamide in the absence of a satisfactory clinical response. Improvement rates after immunotherapy administration are usually modest and most patients continue experiencing seizures (80%) and cognitive impairment (69%) [30]. First-line treatment, in the acute phase, consists of either high-dose corticosteroid therapy (initially IV, then PO), IVIG (2 g/kg for 3–5 days), or plasmapheresis (5–10 sessions every other day). Second-line treatment is considered if, after 2–3 weeks of initiating first-line therapy, there is no response or if the initial symptoms are severe, and it consists of the administration of immunosuppressants (cyclophosphamide, rituximab, tocilizumab, bortezomib, or azathioprine) [27].

A study by Zhang et al. [39] demonstrates that the antibody-associated AE patients who received TPE exhibited some degree of rapid clinical improvement (1–2 months) after an absent response to first-line pharmacotherapy. In conclusion, tumor removal and pharmacotherapy currently remain the first-line treatment in the majority of AE cases, but TPE might be a reasonable option to consider in patients with severe antibody-associated AE with absent or limited improvement after pulse steroids or IVIG.

A review of the literature showed that LE associated with GAD65 antibodies was frequently refractory to standard immunotherapies, and recovery was often incomplete. Observational studies provide the mainstay of evidence guiding first- and second-line immunotherapy administration in AE, and whereas these typically result in some clinical improvements, almost all patients have residual neuropsychiatric deficits, and many experience clinical relapses [40].

Considering that the patient’s symptoms were refractory to administration of IVIG at 20 g per day for five days, TPE was performed (a course of 5 sessions, every other day), with a favorable clinical response. Long-term discharge treatment consisted of risperidone (as it was proven to have a positive effect on behavioral, cognitive, and psychotic symptoms), memantine (as it was shown that it improved the FBI score in FTD patients, and as an NDMA receptor antagonist it protects neurons against glutamatergic excitotoxicity, and may have neuroprotective effects for anti-GAD65 AE) [41], donepezil (as cholinesterase inhibitors improve cognitive impairment to some degree in AE [26], and as it was found that the cholinergic pathways are affected in FTD [42]), and a combination of levodopa and carbidopa (for improving Parkinsonism).

### 3.6. Limitations

Genetic testing for common bvFTD-associated mutations and functional imaging investigations were not available within our hospital setting as part of the routine diagnostic workup. In addition, out-of-pocket genetic testing was not feasible in this case because of the patient’s financial limitations. Another important limitation is that anti-GAD65 antibodies were detected only in serum, at a modest titer, while CSF antibody testing was not conducted. Testing for Epstein–Barr virus was limited to serum IgM, limiting the potential interpretability as a pathogenic trigger. Because the patient continued follow-up care in another county after discharge, we did not have access to subsequent clinical records and were therefore unable to assess longer-term evolution or document whether the psychiatric, cognitive, and motor symptomatology amelioration was maintained or improved after discharge from our hospital.

## 4. Conclusions

Autoimmune encephalitis is a relatively uncommon and difficult-to-manage disorder with a broad range of neuropsychiatric symptomatology. However, this case proved to be significantly more complex due to the possible coexistence of bvFTD, as both disorders have overlapping symptoms, producing a blended phenotype that risks diagnostic delay. This case report highlights the importance of a thorough investigation, emphasizing AE antibody panel investigations and the importance of considering the diagnosis of AE in patients presenting with acute or subacute altered mental status, even in those already diagnosed with a major neurocognitive disorder. Antibody testing should be considered more often, especially if “red flags” are present, and clinicians should have a better understanding of the varied causes of cognitive decline in order to establish a prompt diagnostic approach, allow early initiation of proper treatments, and improve clinical outcomes.

## Figures and Tables

**Table 1:** Chronological summary of the patient’s clinical course.

Clinical Stage	Key Findings/Events	Treatment/Outcome
Emergency department presentation	Acute/subacute confusion, disorientation, short-term memory loss, perseverations, psychomotor retardation, apathy, transient visual hallucinations	Admitted for further neurological workup
Initial neurological assessment	Bradykinesia, symmetric rigidity, camptocormia, positive Romberg, convergence insufficiency, nystagmus, pyramidal signs on the left	Supported an organic neurological process
Workup	Infection not supported; daily glycemic values within normal range, HbA1C normal; mild proteinorachia and glycorrhachia; elevated phosphorylated tau; EEG with asymmetric temporal theta slowing; brain CT shows frontotemporal atrophy with ventricular enlargement	Supported encephalopathy and a possible neurodegenerative contribution
Initial treatment in neurology	Persistent fluctuating neuropsychiatric and motor symptoms; progressive behavioral syndrome plus frontotemporal atrophy and elevated p-tau favored a working diagnosis of bvFTD	IVIG for 5 days, with minimal apparent improvement; transferred to psychiatry for further management
Psychiatric admission	Poor self-care, stereotypies, utilization behavior, psychomotor agitation, slow speech, blunted affect, perseveration, concrete thinking, visual hallucinations, executive dysfunction, impaired attention, absent insight	Symptomatic psychopharmacological treatment, without meaningful overall improvement
Autoimmune re-evaluation	Because of fluctuating course and unfavorable evolution, an AE antibody panel was requested	Anti-GAD65 positive (60 UI/mL), other antibodies negative; autoimmune contribution suspected
Return to neurology/escalation of treatment	Clinical picture considered refractory to IVIG	Therapeutic plasma exchange (5 sessions, every other day) initiated
Response to TPE	After the second session: remission of psychotic symptoms and improvement in cognition and motor function	Marked improvement in gait, posture, rigidity, bradykinesia, praxia, and attention
Post-TPE cognitive reassessment	Partial but incomplete cognitive improvement	MMSE improved from 11/30 to 16/30; MoCA improved from 3/30 to 6/30
Discharge	Stable, with marked motor improvement and remission of psychotic symptoms, but persistent cognitive impairment and need for supervision	Discharged on symptomatic psychiatric/neurological treatment with follow-up recommendation

Abbreviations: bvFTD, behavioral variant frontotemporal dementia; CT, computed tomography; EEG, electroencephalography; GAD65, glutamic acid decarboxylase 65; HbA1C, hemoglobin A1c; IVIG, intravenous immunoglobulin; MMSE, Mini-Mental State Examination; MoCA, Montreal Cognitive Assessment; TPE, therapeutic plasma exchange.

## Data Availability

The original contributions presented in this study are included in the article. Further inquiries can be directed to the corresponding author.

## Abbreviations

The following abbreviations are used in this manuscript:

AE	Autoimmune encephalitis
Anti-GAD65	Anti-glutamic acid decarboxylase 65 antibodies
Anti-VCA	Anti-viral capsid antigen (Epstein–Barr virus serology)
Bid	Twice daily
BvFTD	Behavioral variant frontotemporal dementia
C9ORF72	Chromosome 9 open reading frame 72
CNS	Central nervous system
CRP	C-reactive protein
CSF	Cerebrospinal fluid
CT	Computed tomography
ED	Emergency department
EEG	Electroencephalography
FESR	Erythrocyte sedimentation rate
FBC	Full blood count
FBI	Frontal Behavior Inventory
FTD	Frontotemporal dementia
GABA	Gamma-aminobutyric acid
GAD	Glutamic acid decarboxylase
GAD-AE	GAD65-associated autoimmune encephalitis
IgG	Immunoglobulin G
IV	Intravenous
IVIG	Intravenous immunoglobulin
LE	Limbic encephalitis
MMSE	Mini-Mental State Examination
MoCA	Montreal Cognitive Assessment
MRI	Magnetic resonance imaging
MSE	Mental state examination
NMDA	N-methyl-D-aspartate
nfvPPA	Nonfluent variant primary progressive aphasia
PO	Per os (oral administration)
PPA	Primary progressive aphasia
Q6h/q8h/q12h	Every 6/8/12 h
Qd	Once daily
SPS	Stiff-person syndrome
svPPA	Semantic variant primary progressive aphasia
TPE	Therapeutic plasma exchange
